# Annual acknowledgement of manuscript reviewers

**DOI:** 10.1186/1749-8546-9-5

**Published:** 2014-02-11

**Authors:** Siu-wai Leung

**Affiliations:** 1 International Society for Chinese Medicine, Macao, China

## Abstract

**Contributing reviewers:**

The editors of *Chinese Medicine* would like to thank all our reviewers who have contributed to the journal in Volume 8 (2013).

## 

James Adams

United States of America

Nicolino Ambrosino

Italy

Vasumathi Asha

India

Marcio Babinski

Brazil

Zhaoxiang Bian

Hong Kong

Rai Chi Chan

Taiwan

Bei-Hung Chang

United States of America

Raymond Chuen-Chung Chang

Hong Kong

Calvin Yu-Chian Chen

United States of America

Chieh-Fu Chen

Taiwan

Wei Chen

China

Chung-wah Cheng

Hong Kong

Veeramani Chinnadurai

Saudi Arabia

William CS Cho

Hong Kong

Mei Chong

Canada

Selvakumar Elangovan

India

Yibin Feng

Hong Kong

Arthur Sa Ferreira

Brazil

Johannes Fleckenstein

Germany

Ko Fujimori

Japan

Krishnamoorthy Ganesan

India

Song Gao

United States of America

Hong-You Ge

Denmark

Ilko Getov

Bulgaria

Marilena Gilca

Romania

Anthony Good

Australia

Xinfeng Guo

China

Sang-Bae Han

South Korea

Shanshan He

United States of America

Morag Heirs

United Kingdom

Janice Yuen-Shan Ho

Hong Kong

Tingjun Hou

China

Hao Hu

Macao

Dejian Huang

Singapore

Yi-Tsau Huang

Taiwan

Shinn-Jang Hwang

Taiwan

Seung-Il Jeong

South Korea

Lin Jiang

United States of America

Hsueh-Fen Juan

Taiwan

Kyung-Hwan Jung

South Korea

Md Asaduzzaman Khan

China

Jae Kim

South Korea

Kam Ming Ko

China

Jun Kong

China

Victor Kuete

Cameroon

Jean-Marc Lavoie

Canada

Myeong Soo Lee

South Korea

Kelvin Leung

Hong Kong

Siu-wai Leung

Macao

Chien-Chun Li

Taiwan

Jian Li

China

Shao Li

China

Xing-Tai Li

China

Zhixiu Lin

Hong Kong

Ulrike Lindequist

Germany

Zhanguang Liu

China

José María Moreno-Navarrete

Spain

Debra Moriarity

United States of America

Takayuki Nakagomi

Japan

Tzi Ng

Hong Kong

Kyoko Ohashi

United States of America

Satoshi Onodera

Japan

Gerald Pang

Australia

Can Peng

United States of America

Yong Peng

China

Zengfu Peng

China

Santiago Perez-Lloret

France

Rongbiao Pi

China

Raghu Rajasekaran

Taiwan

Kannan Ramamirthum

India

Daniel Royse

United States of America

Vitoon Saengsirisuwan

Thailand

K Satyamoorthy

India

Subhabrata Sengupta

India

Jiangang Shen

Hong Kong

Luan Shu

China

Jingyuan Song

China

Mark Stoeckle

United States of America

Keisuke Suzuki

Japan

Stephen Cho Wing Sze

Hong Kong

Ivan Tancevski

Austria

Margaret Diana van Die

Australia

Seda Vatansever

Turkey

Howard Vernon

Canada

Sivarama Prasad Vinjamury

United States of America

Jinn-Chyi Wang

Taiwan

Fang Wei

China

Darong Wu

China

Taixiang Wu

China

Liwei Xie

United States of America

Ru Yan

China

Jianping Ye

United States of America

Hw Yeung

Macao

Guohua Yin

United States of America

Jia Yongliang

China

Ken Yung

Hong Kong

Yusmazura Zakaria

Malaysia

Zhen Zhan

China

Junhua Zhang

China

Nevin L Zhang

China

Zhen Zheng

Australia

